# scMINA: an interpretable workflow for paired scRNA–scATAC multiome integration and regulatory prioritization

**DOI:** 10.1093/nargab/lqag140

**Published:** 2026-11-10

**Authors:** Shuwen Zhang, Hongru Hu, Xue Wang, Chengjie Xiong, Takahisa Kanekiyo, Yan W Asmann, Yingxue Ren

**Affiliations:** Department of Quantitative Health Sciences, Mayo Clinic, Rochester, MN 55905, United States; Integrative Genetics and Genomics, University of California, Davis, CA 95616, United States; Department of Quantitative Health Sciences, Mayo Clinic, Jacksonville, FL 32224, United States; Division of Biostatistics, Washington University School of Medicine, St. Louis, MO 63110, United States; Department of Neuroscience, Mayo Clinic, Jacksonville, FL 32224, United States; Department of Quantitative Health Sciences, Mayo Clinic, Jacksonville, FL 32224, United States; Department of Quantitative Health Sciences, Mayo Clinic, Jacksonville, FL 32224, United States

## Abstract

Single-cell multiomic technologies that jointly profile transcriptomic and epigenomic states provide unprecedented opportunities to investigate cellular heterogeneity and gene regulatory mechanisms in complex diseases. However, analysis of paired single-cell RNA-seq (scRNA-seq) and chromatin accessibility (scATAC-seq) data remains computationally challenging due to modality-specific technical variation, integration complexity, and limited interpretability of downstream regulatory inference. Here, we present scMINA (single-cell Multimodal Integration and Network Attribution), a modular and interpretable analytical workflow for the analysis of paired single-cell multiome datasets. scMINA comprises coordinated modules for multimodal integration, technical covariate correction, and regulatory feature prioritization, enabling systematic characterization of cellular states across transcriptomic and epigenomic modalities. In addition, scMINA incorporates explainable artificial intelligence-based attribution strategies to prioritize candidate genes and transcription factors associated with cell-state heterogeneity and disease-related regulatory alterations. We applied scMINA to the single-cell multiome data from the Seattle Alzheimer’s Disease Brain Cell Atlas, where the workflow robustly resolved major glial cell populations and identified regulatory features associated with Alzheimer’s disease pathology. By coupling multimodal integration with interpretable regulatory prioritization, scMINA provides a practical workflow for systematic analysis and biological interpretation of paired scRNA-seq and scATAC-seq datasets.

## Introduction

Understanding complex diseases at the cellular level requires dissecting both gene expression programs and their regulatory mechanisms. While single-cell RNA sequencing (scRNA-seq) reveals the transcriptional landscape of individual cells [1] , single-cell Assay for Transposase-Accessible Chromatin (scATAC-seq) uncovers chromatin accessibility patterns that shape gene activity [2, 3]. Together, these technologies, especially the recent joint profiling assays [4, 5], provide holistic views of cell states and gene regulation, where researchers are able to trace how transcription factors, *cis*-regulatory elements, and promoters coordinate to control cell state dynamics [3, 6]. Despite their potential, integrating multiomic data remains challenging due to inherent differences in data structure, distinct feature types, sparsity levels, and susceptibility to batch effects. Moreover, linking chromatin accessibility to downstream transcriptional expression on a cell-by-cell basis is nontrivial, as regulatory relationships are often context-dependent and cell state-specific. Unlike scRNA-seq analysis alone where established best practices and analysis pipelines are widely adopted [7, 8], multiomic data integration and analysis lack standardized workflows, making it difficult to extract biologically meaningful insights and limiting their applications to disease studies.

The analytic workflow for single cell multiomics faces several key challenges. One essential step in processing scATAC-seq data is to ensure that chromatin accessibility features are represented in a unified feature space. However, the cell-by-peak matrices generated from individual samples often contain non-overlapping, sample-specific peaks, requiring a unified peak calling step across samples to harmonize the feature space and enable joint analysis of chromatin accessibility data. Once paired cell-by-feature matrices are constructed from scRNA-seq and scATAC-seq modalities, the challenge shifts to integration. Broadly, two classes of computational methods are commonly used for multiomic integration. The first includes matrix factorization approaches such as MOFA [9] and MOFA+ [10], which decompose input data into shared and modality-specific latent factors. These methods are interpretable but are generally limited to capturing linear relationships and not scalable to handle full feature sets. The second class involves deep learning-based representation learning approaches, which can model complex, non-linear interactions between features across modalities and flexibly incorporate confounding variables such as batch effects. Among these, unsupervised generative models such as MultiVI [11] uses variational autoencoders to infer joint latent representations, while supervised models such as scPair directly link chromatin accessibility to gene expression through paired training [12]. These approaches aim to learn biologically meaningful cell state embeddings that serve as a foundation for downstream analyses. However, embeddings derived from deep learning models can be more difficult to interpret directly.

For downstream analysis, different strategies exist with their own advantages and drawbacks. RNA-based analyses can be used to identify marker genes for major cell types, though they may lack insight into regulatory mechanisms. For example, conventional methods such as Seurat [7, 8] and Signac [13] can compute gene activity scores based on proximal chromatin peaks. However, these scores often rely on the distance between a peak and a transcription start site, potentially missing distal regulatory interactions. Correlation-based methods, such as Functional Inference of Gene Regulation (FigR) [14], on the other hand, can model relationships between gene expression and chromatin accessibility simultaneously, enabling the reconstruction of gene regulatory networks (GRNs) and identification of Transcription Factor-Domains of Regulatory Chromatin (TF-DORC) associations. Finally, deep learning models offer powerful embeddings for integrated cell state representation, but their complexity can obscure biological interpretability. A potential solution is to apply feature attribution techniques, including gradient-based approaches such as Integrated Gradients [15], to the learned models to prioritize input features (e.g. genes, peaks) that contribute most to cell state definitions. This layer of interpretability bridges the gap between high-performing black-box models and biologically actionable insights.

To overcome these challenges, we developed scMINA (single-cell multimodal integration and network attribution), a comprehensive and interpretable workflow for the integration and analysis of single-cell multiome data. scMINA systematically addresses key challenges in multiomic analysis, from unified peak calling and modality alignment to the reconstruction of gene regulatory networks and model-based interpretability. By benchmarking both factorization- and deep learning-based integration methods, scMINA identifies optimal strategies for learning unified cell state representations. These embeddings serve as a foundation for downstream analyses, including differential gene expression, transcription factor-target inference, and feature attribution using explainable artificial intelligence (XAI). Together, scMINA enables a unified, scalable, and biologically grounded approach to deciphering cell states and regulatory programs from complex multiomic datasets.

To demonstrate the utility of the scMINA workflow, we applied it to paired single-cell multiomic data from the Seattle Alzheimer’s Disease Brain Cell Atlas (SEA-AD) [16] and focused our analysis on glial cells in the middle temporal gyrus (MTG), a brain region known to be affected early in Alzheimer’s disease (AD). scMINA revealed AD-associated shifts in glial subpopulations, distinct transcriptional and epigenomic signatures, and regulatory networks that connected key transcription factors to target genes. Notably, the implementation of interpretable machine learning methods allowed us to identify genes with strong contributions to disease-related chromatin accessibility in specific glial subtypes. These results highlight scMINA’s capacity to uncover biologically meaningful and mechanistically informative insights from complex single-cell multiomic datasets.

## Materials and methods

### Dataset description

We obtained the SEA-AD dataset for the MTG from two sources (accessed 25 September 2023): the multiome data files from Synapse (ID: syn29879329) and the processed RNA expression matrix from Chan Zuckerberg CELL x GENE (https://cellxgene.cziscience.com/collections/1ca90a2d-2943-483d-b678-b809bf464c30). The initial dataset contained 98 601 cells from 16 normal donors and 52 755 cells from 12 dementia donors.

### Sample selection and data processing for scMultiome integration benchmarking

To benchmark scMultiome integration methods, we selected four donors to ensure balanced representation across conditions and biological sexes while maximizing cell numbers. These donors included H20.33.016 (female with dementia, 8130 cells), H20.33.017 (male with dementia, 6797 cells), H20.33.008 (female normal, 8414 cells), and H20.33.025 (male normal, 8950 cells).

For scATAC-seq data processing, we first conducted peak calling using MACS2 on ATAC fragments from these four donors using the HG38 reference genome. After excluding non-autosome peaks, we identified 190 133 peaks. We then filtered peak regions opening in less than 1% of cells per individual and kept the intersection set across all four donors, resulting in 151 859 peak features across 32 291 cells. We further filtered cells to include only those with 300–30 000 peak features, resulting in 30 090 cells for downstream analysis. To process scRNA-seq data, we removed genes present in less than 1% of cells per individual. Taking the intersection set across all four donors yielded 19 400 gene features. We then filtered cells to include only those with 1000–150 000 gene features, resulting in 32 289 cells for downstream analysis.

### Benchmarking scMultiome integration methods

We collected the intersection of 30 089 cells present in both data modalities, and split them into a modeling set containing 24 072 cells (80%) and a held-out test set of 6017 cells (20%). The modeling set was further divided into 19 258 cells (80%) for training and 4814 (20%) cells for validation to enable early stopping and prevent overfitting. To meet modeling requirements using MultiVI and scPair, we converted ATAC peak counts to binary values.

For scPair execution, we followed the tutorial guidelines available on GitHub (https://github.com/quon-titative-biology/scPair/tree/main/tutorials). The ATAC to RNA component utilized two hidden layers with 900 and 30 nodes, respectively, and a latent dimension of 30. We set the learning rate to 0.001, batch size to 300, and employed batch normalization and layer normalization with GELU activation function. We used the Adam optimizer and set the dropout rate to 0.1, and. For the output, we implemented the zero-inflated negative binomial distribution (ZINB) for RNA counts. The RNA to ATAC component maintained similar settings but employed the Bernoulli distribution for the binarized peaks. Sex, donor ID, and disease status were included as covariates using one-hot encoding during model training. The mapping networks were implemented as linear transformations.

We ran MultiVI using scvi-tools and followed the original paper [17] by not explicitly incorporating the modality penalty term in the loss function. We designated donor ID as the “batch_key” and set the latent dimension as 30 while keeping other parameters at their default values as specified in the tutorial: https://docs.scvi-tools.org/en/stable/tutorials/notebooks/multimodal/MultiVI_tutorial.html.

For MOFA+ testing, we first preprocessed the data using the muon package (https://muon.scverse.org). The RNA modality underwent library size normalization with a size factor of 10 000, followed by log_1_p transformation for variance stabilization. We then selected highly variable genes using the setting of flavor = “seurat,” resulting in 3606 genes. For the ATAC modality, we applied the “Term Frequency Inverse Document Frequency of records” transformation with a scale factor of 10 000 and performed feature selection using the setting of min_mean = 0.05, max_mean = 1.5, min_disp = 0.5 as suggested in the tutorial: https://muon-tutorials.readthedocs.io/en/latest/single-cell-rna-atac/pbmc10k/2-Chromatin-Accessibility-Processing.html. We also utilized mofapy2 (https://github.com/bioFAM/mofapy2), with 30 factors (K = 30), enabling ard_weights and ard_factors, and employed GPU acceleration with “fast” convergence mode to optimize processing time. Afterward, we utilized mofaX (https://github.com/bioFAM/mofax) to extract the variables of loadings and factors and fetch the values of 30-dimensional representations.

### Benchmark performance evaluation

To evaluate performances of the three integration methods, we first investigated cell representation quality using classical measures, including cell clustering annotation accuracy by Adjusted Rand Index (ARI) [18] and Normalized Mutual Information (NMI) [19], cell type separation by Average Silhouette Width (ASW) [20] and Cell-specific Local Inverse Simpson’s Index (cLISI) [21], and batch mixing by batch-specific Average Silhouette Width (batchASW) [22].

We further assessed cross-modality alignment using Fraction of Samples Closer than True Match (FOSCTTM) [23]. For cross-modality prediction, we employed multiple complementary metrics. For ATAC-to-RNA prediction, we used the Pearson Correlation Coefficient (PCC) and Spearman Correlation Coefficient (SCC) for RNA prediction, and Area Under ROC Curve (auROC) and 1-Binary Cross Entropy (1-BCE) for ATAC prediction.

### Selection of scPair for scMultiome integration

scPair is implemented as the default integration option in scMINA based on its previously demonstrated performance across diverse paired scRNA-seq/scATAC-seq datasets and its performance in the SEA-AD analysis presented here. In the original scPair study, scPair was systematically evaluated across seven paired scRNA-seq/scATAC-seq datasets spanning multiple tissues and species and demonstrated strong performance relative to existing multimodal integration approaches. Its performance has also been independently evaluated [24], which further demonstrated the competitive performance of scPair across diverse datasets. Together with the SEA-AD analysis presented here, these results provide support for its use as the default integration option in scMINA.

The selection of scPair as the default does not imply that it is universally optimal for all multiome datasets. scMINA is designed as a modular workflow, allowing users to substitute alternative integration approaches according to dataset characteristics and analytical objectives. Users with previously integrated and annotated datasets may also bypass the integration module and proceed directly to downstream scMINA analyses.

### scMultiome integration for glial cells using scPair

We applied scPair on 26 of the 28 donors from the SEA-AD MTG multiome dataset focusing on glial cells, excluding H20.33.040 and H20.33.043 due to their relatively low glial cell counts (48 and 416 cells, respectively). The remaining donors each contributed more than 1000 cells. From scPair-learning embeddings, we obtained 25 054 cells, 104 140 peaks, and 18 088 genes. We also performed separate peak calling for each glial cell types (microglia, astrocytes, oligodendrocytes, and OPC) to ensure that we capture cell type-specific peaks for cell-population-specific scPair modeling. The training settings were consistent with the benchmarking experiments.

### Data normalization, cell clustering, and differential expression analysis

Seurat (v5.0.0) [8] was employed for all downstream analyses, starting with global-scaling normalization using the “LogNormalize” method, followed by log-transformation for downstream analyses. Highly variable features (default: 3000 genes) were identified for each sample using the “FindVariableFeatures” function to highlight biological signals. Linear transformation was applied to the combined dataset as a standard pre-processing step. Harmony [21] was used for batch correction and primary clustering based on scPair-derived representations. Principal component analysis was conducted for dimensionality reduction, and the Uniform Manifold Approximation and Projection (UMAP) [25] method was used to visualize the integrated dataset. The “FindNeighbors” and “FindClusters” functions were used for cell clustering, with cluster-specific markers identified using the “FindConservedMarkers” function. Clusters were annotated to known cell types based on marker genes. Differentially expressed genes (DEGs) between AD and controls were identified using the MAST method implemented in Seurat, with a significance threshold of adjusted *P* < .05 and |log_2_ fold change| > 1. Visualization of marker genes and DEGs was conducted using the FeaturePlot and VlnPlot functions in Seurat alongside the ggplot2 package (https://ggplot2.tidyverse.org). Circos plots of overlapping DEGs among different cell type groups were generated using Metascape [26]. The enrichR package [27] was used to perform functional enrichment analysis of DEGs using the Gene Ontology Biological Process (GO-BP) gene set.

### Glial cell subpopulation analysis and feature attribution calculations

Each glial cell types was re-clustered to reveal subpopulations using the same Seurat procedures as previously described. For scPair model interpretation, we employed two widely used feature attribution approaches in single cell studies: Integrated Gradients implemented by Captum package (https://captum.ai) [15] and Path-Explainer from PAUSE (https://github.com/suinleelab/PAUSE) [28]. We used cluster-specific averaged input values as the baseline for feature attribution, calculating, and averaging attribution scores within clusters. After selecting the top 100 highly attributed features per method per cluster, we identified highly-contributing features through intersection across methods and clusters.

### Chromatin opening visualization

For ATAC chromatin opening visualization on the genome, we used the function CoveragePlot() from Signac (https://stuartlab.org/signac/) [13] with selected chromatin regions and corresponding genes.

### Domain of regulatory chromatin analysis

We employed the FigR [14] framework to interrogate the scMultiome data for the inference of GRNs. *Cis*-regulatory associations were assessed by calculating gene-peak correlations, followed by the definition of domains of regulatory chromatin (DORCs), representing loci where chromatin accessibility and gene expression are tightly coupled. The core regulatory interactions were then inferred through the FigR framework’s GRN inference function, which integrates DORCs with scRNA-seq data to identify transcription factors (TFs) likely to regulate target genes. The identified TF-gene pairs were filtered by statistical significance, and the resulting GRN was subjected to network analysis and functional enrichment.

### Workflow implementation and reproducibility

The scMINA computational workflow was implemented using Nextflow DSL2 [29, 30] to facilitate reproducible and scalable execution of the analysis. The workflow consists of modular processes for scPair model training, embedding inference, clustering, feature attribution, integration of scPair embeddings with Seurat, and downstream gene regulatory network analysis using FigR. Individual modules can also be executed independently depending on the analysis requirements. Software dependencies for the workflow were managed using Conda, with package versions specified in the environment configuration file.

To improve reproducibility of stochastic computational procedures, random seeds were fixed where supported by the corresponding software. The same random seed and parameter settings were used across the reported analyses unless otherwise specified. For the machine learning and feature-attribution analyses, model architecture, training parameters, input data partitions, and attribution settings were maintained consistently across analyses to reduce variability arising from model initialization, data sampling, and optimization. Relevant parameters for each analysis step are specified in the corresponding workflow and analysis scripts. The complete Nextflow workflows, configuration files, software environment, and supporting Python and R scripts are available through the scMINA GitHub repository.

## Results

### scMINA overview

scMINA is a comprehensive computational workflow that enables robust data integration, biological interpretation, and mechanistic insight through explainable machine learning (Fig. 1). The workflow begins with multimodal data processing and unified feature construction, followed by multimodal data integration to learn unified cell state representations. The final stage involves downstream analysis through complementary strategies for *in silico* cross-validation, including conventional bioinformatics analysis, correlation-based regulatory inference, and attribution-based model interpretability.

**Figure 1.**
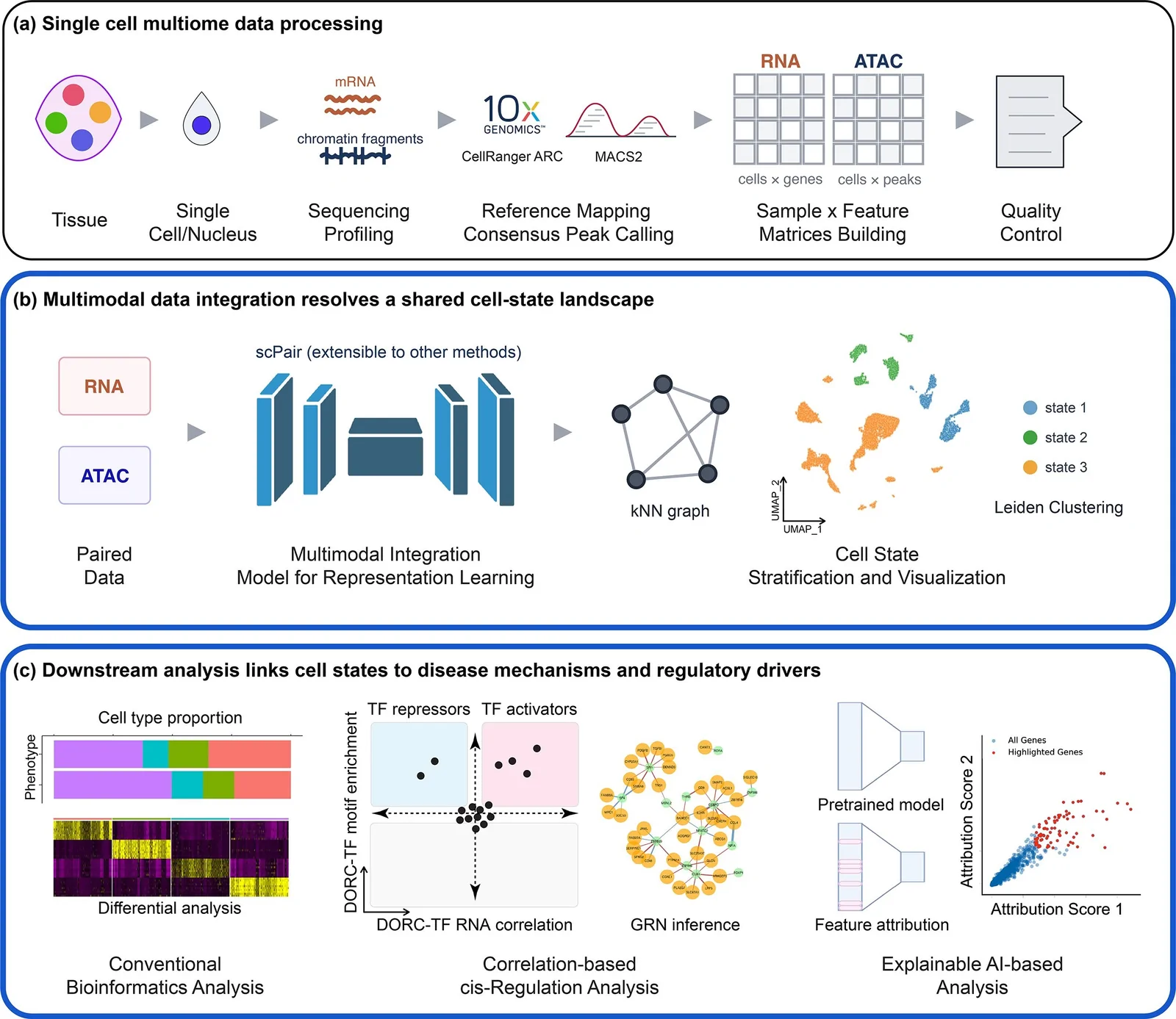
Overview of the scMINA workflow for single cell multiomic data integration and interpretable analysis. (**a**) Data processing workflow: Raw sequencing data from single-cell multiomics assays generate paired transcriptomic and epigenomic profiles from the same cells. These data are processed through reference mapping, unified peak calling, and the construction of gene and peak count matrices that serve as input for downstream scMINA analysis. (**b**) Multimodal integration: The scPair method is selected to learn unified cell state representations, which are further used for clustering and annotation. (**c**) Downstream analysis: the workflow supports three parallel downstream modules: (i) conventional analysis including cell type proportion and differential expression testing, (ii) correlation-based *cis-*regulatory analysis such as TF-DORC associations and gene regulatory network inference using FigR, and (iii) explainable AI-based analysis using pretrained representation learning models to prioritize gene and TF signatures via feature attribution. The blue outlines indicate components included in the scMINA workflow.

scMINA is designed for paired single-cell multiomic datasets that jointly profile chromatin accessibility (scATAC-seq) and gene expression (scRNA-seq) from the same cells. Prior to applying the scMINA workflow, raw sequencing data are typically processed using the 10x Genomics Cell Ranger ARC pipeline to generate cell-by-feature count matrices. To harmonize chromatin features across samples, unified peak calling can be performed using tools such as MACS2 [31] to obtain a consistent peak set and corresponding cell-by-gene and cell-by-peak count matrices. Standard quality control procedures with tunable parameters are then applied to retain high-confidence cells and features, providing reliable inputs for downstream scMINA analysis. (Fig. 1a).

To support the integration of paired scRNA-seq and scATAC-seq data, scMINA incorporates scPair as the default integration method, based on our systematic benchmarking of multiple computational approaches (Fig. 1b), including the matrix factorization methods such as MOFA+ [10], and deep learning-based approaches such as MultiVI [11] and scPair [12]. The comparative performance of these methods across modality integration, batch effect removal, and preservation of biological structure is presented in detail in the following section, “Benchmarking single-cell multiomics integration methods.” Based on these benchmarking results, we selected scPair-derived cell embeddings for the subsequent analyses performed in this study.

Importantly, scMINA is designed as a modular and flexible workflow and does not require the exclusive use of scPair. Users may substitute alternative integration methods according to their datasets or analytical objectives, with checkpoints provided between major analytical stages. For example, users with previously integrated and annotated data, generated through their own analyses or obtained from public resources, can bypass the integration stage and proceed directly to downstream analyses.

The scMINA workflow supports a suite of downstream modules designed to extract mechanistic and regulatory insights from the integrated cell state space (Fig. 1c). These include three parallel analysis branches. The first branch involves conventional analyses supported through established tools such as Seurat [7, 8] and Signac [13] to identify differentially expressed genes and accessible peaks across cell types or phenotypic groups. The second branch deploys FigR [14], a correlation-based approach that infers *cis-*regulatory associations between gene expression and chromatin accessibility to reconstruct gene regulatory networks. This enables the identification of TF-DORC relationships and the assembly of cell type-specific GRNs. The third branch incorporates feature attribution methods that quantify the contribution of individual features to cell state discrimination to enhance interpretability of the learned embeddings. Specifically, we used Integrated Gradients (via Captum) [15] and Path-Explainer (from PAUSE) [28] to assign attribution scores to input features. These methods allow the users to prioritize genes and regulatory elements with strong influence on modality alignment and integration. By integrating these attribution results with outputs from FigR and conventional differential analyses, users can identify convergent regulatory signatures. The XAI module in scMINA is also designed to be modular and extensible. For example, users may incorporate external biological knowledge or gene modules inferred from pretrained models such as sciLaMA [32], which leverages large language models to learn contextual gene representations. This added flexibility enables multi-scale interpretability across diverse data types and use cases.

### Benchmarking single cell multiomics integration methods

While the preprocessing pipeline for single-cell multiomic data has become increasingly standardized, integration remains a critical and customizable step that heavily influences downstream biological interpretation. In particular, aligning transcriptomic and epigenomic modalities into a unified representation requires careful method selection, as performance varies depending on dataset structure, modality balance, and analytical objectives. To identify the most suitable integration strategy for use within the scMINA workflow, we compared three representative methods: scPair, MultiVI, and MOFA+, each corresponding to a distinct class of integration models as described earlier. Factorization-based models, such as MOFA+ [9, 10], decompose multiomic data into shared factors, making them useful for identifying biologically relevant variation. Deep learning-based approaches introduce more flexible, data-driven strategies for aligning different modalities. Among these, MultiVI [11] applies unsupervised variational autoencoders for cross-modality alignment, while scPair [12] implements a supervised learning approach that links chromatin accessibility and gene expression end to end. All three methods are designed to learn low-dimensional embeddings that integrate information across modalities and enable discovery of coherent cellular states.

As a representative case study, we evaluated integration performance on a subset of four samples from the SEA-AD single-cell multiome dataset. These samples were selected based on their high cell counts and balanced representation across biological sex and Alzheimer’s disease status. We focused our evaluation on the quality of cell state representation learning, using a suite of established integration metrics to assess clustering performance, cell type separability, and batch effect correction (Fig. 2a). Clustering accuracy was measured using ARI and NMI, while cell type separation was evaluated using ASW and cLISI. To quantify batch effect removal or batch mixing while preserving biologically meaningful signals, we used batch-specific ASW and iLISI.

**Figure 2.**
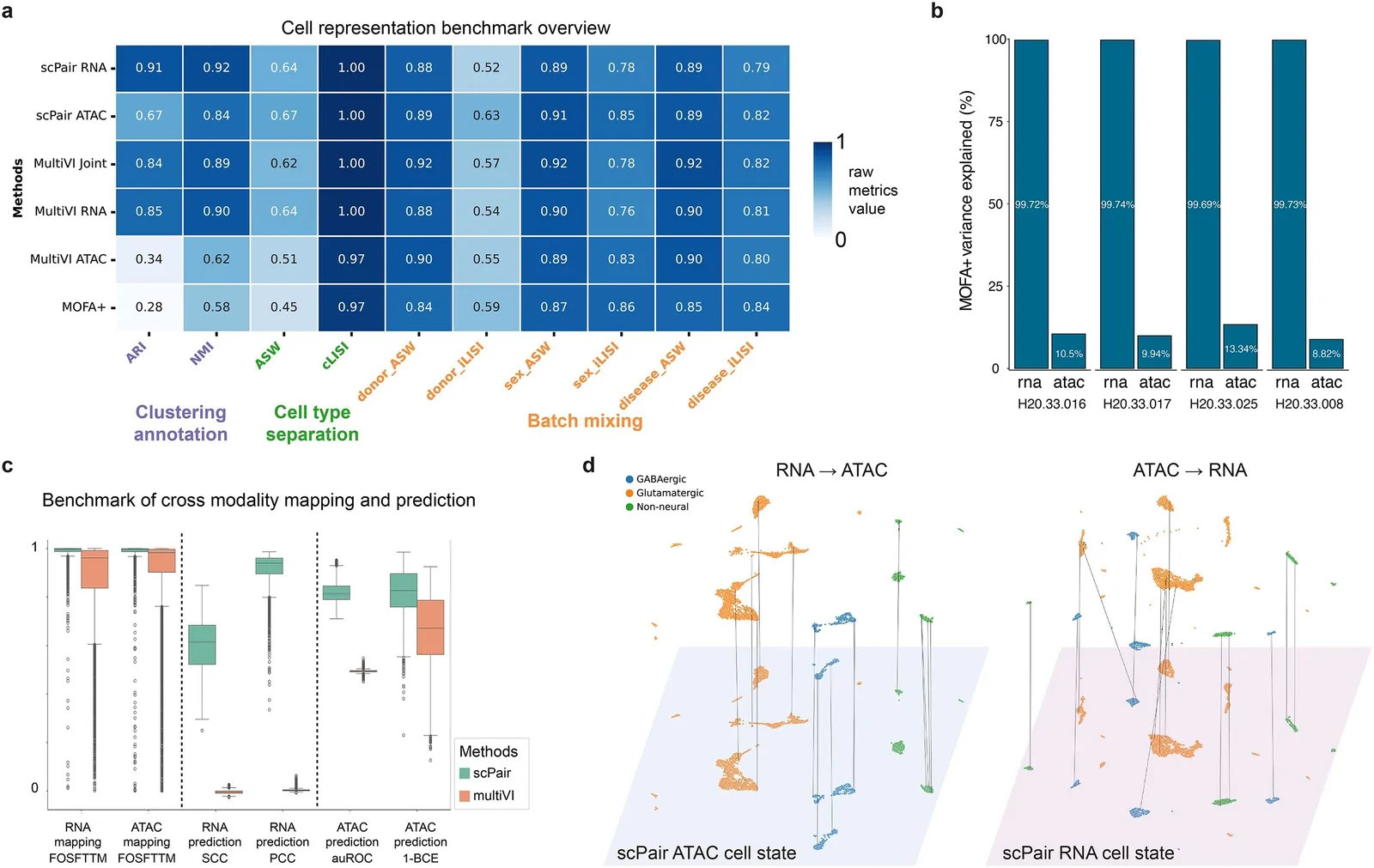
Benchmarking of scMultiome test data using scPair, MultiVI and MOFA+ . (**a**) Evaluation of cell representation generated by scPair, MultiVI, and MOFA+, displaying each method’s ranking and score across the different evaluation criteria. Methods are evaluated based on their capacity for clustering annotation (measured by ARI and NMI), cell type separation (ASW and cLISI), and batch mixing (covariate-specific ASW and iLISI metrics). Higher scores indicate better performance. (**b**) Percentage of variance exampled by RNA and ATAC using MOFA+ in each of the four testing samples. (**c**) Performance benchmarking of scPair and MultiVI across cross-modality mapping tasks, with evaluation metrics calculated on held-out test sets. Box plots display performance metrics including 1-Fraction of Samples Closer Than the True Match (1-FOSCTTM), SCC, PCC, auROC, and 1-Binary Cross Entropy (1-BCE), where higher values indicate better performance. In the box plots, the minima, maxima, centerline, bounds of box, and whiskers represent the minimum value in the data, maximum, median, upper and lower quartiles, and 1.5× interquartile range, respectively. (**d**) Left: UMAP visualization of the ATAC-based (ground truth, bottom) and RNA-to-ATAC (mapped, top) cell state spaces as learned by scPair using the held-out test set. Each point represents a cell, with lines connecting each cell’s observed ATAC state and mapped ATAC state (derived from RNA state). Colors indicate major cell classes based on original study annotations. Right: An analogous UMAP for RNA-based (ground truth, bottom) and ATAC-to-RNA (mapped, top) cell states, also learned by scPair using the same held-out test set.

Among the three methods, scPair ranked highest across most integration metrics, followed by MultiVI, with MOFA+ ranking lowest in most cases (Fig. 2a). Although MOFA+ is designed to be an interpretable factorization method, its integration was biased toward RNA and led to underutilization of chromatin accessibility information (Fig. 2b). In contrast, scPair and MultiVI generated separate embeddings for RNA and ATAC and have objectives of predicting or reconstructing the modality-specific features for each cell, which allows for a more balanced flow of modality-specific information.

Cross-modal alignment and prediction are also critical functionalities of a tool for linking gene expression (RNA) to chromatin accessibility (ATAC). Unlike scPair and MultiVI, MOFA+ lacks key capabilities such as modality-specific embedding inference and cross-modality imputation. This means that scPair and MultiVI are able to predict missing modality data after training while MOFA+ cannot. Given these limitations, we directly compared scPair and MultiVI in cross-modality alignment and prediction tasks. To evaluate alignment performance, we measured 1-FOSCTTM and found that scPair outperformed MultiVI (Fig. 2c). For ATAC-to-RNA prediction, we used SCC and PCC to evaluate rank-based and linear correspondence between predicted and observed gene-expression profiles, respectively. These correlation-based metrics were selected to assess preservation of relative expression patterns without requiring exact agreement in raw count magnitudes. For RNA-to-ATAC prediction, auROC and 1-BCE were used to account for the sparse and effectively binary nature of chromatin accessibility data. (Fig. 2c).

Overall, our results demonstrated that scPair was the most effective integration tool for this dataset, outperforming the alternatives in cell clustering, batch effect correction, and cross-modality prediction accuracy. Based on this comprehensive evaluation, the scMINA workflow prioritizes scPair as the default integration strategy and provides output of key quantitative metrics to guide users in assessing integration quality for their own datasets. Using the low-dimensional embeddings learned by scPair, we were able to achieve accurate cross-modal mapping of cell states between RNA and ATAC profiles. This enabled clear resolution of major neuronal and non-neuronal cell types (Fig. 2d). The resulting unified cell state representations, along with their associated visualizations and model checkpoints, are stored as intermediate outputs within the scMINA workflow to facilitate downstream analyses, including regulatory network reconstruction and embedding-based interpretability. While scPair-based integration is the default entry point in scMINA, the workflow retains modular flexibility, allowing users to substitute alternative integration tools according to their specific data characteristics or analysis objectives.

### Conventional downstream analysis workflow reveals glial cell diversity and cell type-specific transcriptome dysregulation in AD

To evaluate the scalability and biological utility of the scMINA workflow, we applied it to the full SEA-AD scMultiome dataset, which includes paired gene expression and chromatin accessibility profiles from over 150 000 nuclei across 28 donors. The cohort encompasses both AD and control samples, balanced by biological sex and other metadata. Given the increasingly recognized roles of glial cells in the structural maintenance and dynamic modulation of neural circuits, as well as in the pathogenesis of AD [33–35], we focused our conventional downstream analyses on glial populations within the MTG.

Using scPair for integration, we analyzed the multiomic profiles from 26 SEA-AD donors with at least 1000 glial cells each (Materials and methods). This approach successfully recaptured major glial cell types in MTG, including astrocytes, microglia, oligodendrocytes, and oligodendrocyte precursor cells (OPCs) (Fig. 3a). These cell types were confirmed by the distinct expression patterns of classical marker genes, including *AQP4* for astrocytes, *CSF1R* for microglia, *MOBP* for oligodendrocytes, and *PDGFRA* for OPCs (Fig. 3b). As part of the scMINA workflow, the corresponding marker gene heatmaps and gene lists are automatically generated and exported for any user-provided clustering or cell type annotations. The chromatin accessibility profiles from the ATAC component further confirmed open chromatin regions near these marker genes (Fig. 3c), which reinforces cell type-specific regulatory landscapes. These region-level accessibility tracks are generated and visualized automatically for each marker gene based on the user-provided annotations as well.

**Figure 3.**
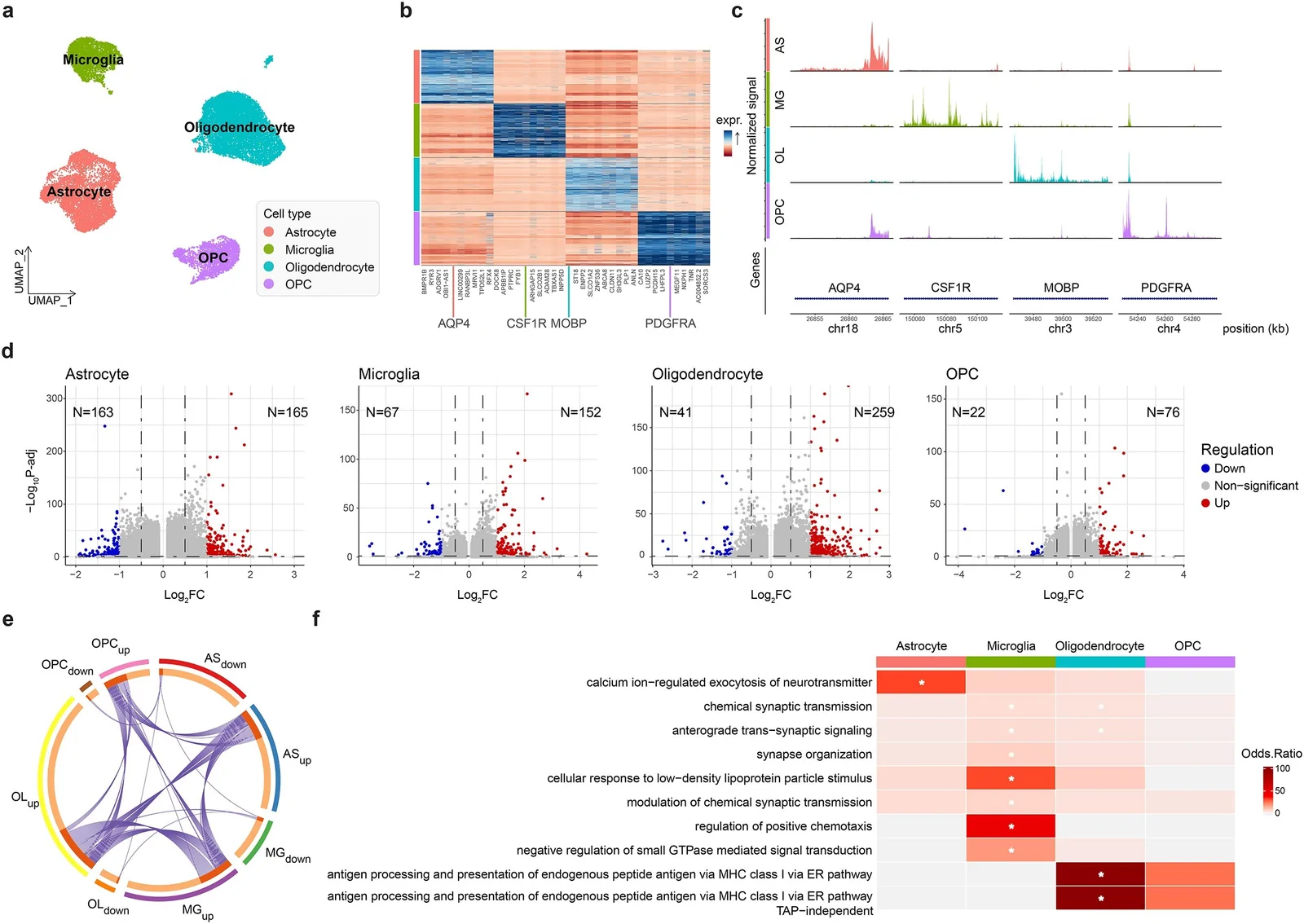
Conventional downstream analysis of SEA-AD glial multiome data. (**a**) UMAP visualization of cell state space learned by scPair, with each color representing a distinct cell type (Astrocytes, Microglia, Oligodendrocytess, OPCs). (**b**) Row-normalized expression heatmap of cell type-specific markers based on the RNA component of SEA-AD multiome data, highlighting marker gene expression profiles across different cell types. (**c**) Chromatin accessibility across cell types visualized for major glial cell type marker genes highlighted in (b) based on the ATAC component of SEA-AD multiome data, illustrating accessible regions near marker genes in different cell populations. (**d**) Volcano plots showing DEG patterns in AD versus controls across major glial cell types. Points represent genes, with “down” (blue) indicating genes downregulated in AD, and “up” (red) for genes upregulated in AD. (**e**) Circos plot showing overlapping DEGs among different cell type groups, identifying shared dysregulated genes across cell populations. (**f**) Heatmap of the odds ratio (quantifying the strength of association between each cell type and the corresponding enriched Gene Ontology terms) for the top GO-BP terms identified from upregulated DEGs in each cell type. DEGs were identified directly from the single-cell differential expression analysis using the MAST method. Asterisks indicate significantly enriched GO terms (adjusted *P* < .01).

Given the disease conditions, we next performed DGE analysis within each glial cell type to identify transcriptomic alterations associated with AD. For each comparison, scMINA outputs include volcano plots, ranked gene lists, and statistical summaries (e.g. log fold-change, adjusted *P*-values), enabling both visualization and downstream reporting. In this case study, compared AD to normal samples, we identified 165 upregulated and 163 downregulated genes in astrocyte, 152 upregulated and 67 downregulated genes in microglia, 259 upregulated and 41 downregulated genes in oligodendrocyte, and 76 upregulated and 22 downregulated genes in OPC (Fig. 3d).

Furthermore, scMINA enables cross-comparison of DEGs across cell types to identify shared transcriptional alterations. In this case study, we found that a substantial number of AD-upregulated genes were shared among multiple glial populations, suggesting the presence of common pathological mechanisms contributing to glial dysfunction in AD (Fig. 3e). To further investigate the functional relevance of these shared DEGs, scMINA performed pathway enrichment analysis for each glial subtype using the corresponding DEG sets. The results revealed distinct but biologically interconnected pathways affected across glial cell types. For instance, astrocytes showed significant upregulation of calcium ion-regulated exocytosis of neurotransmitters pathway, which suggests potential alteration in neurotransmitter release and synaptic function. Microglia had the largest number of upregulated pathways, mostly related to synaptic functions, including chemical synaptic transmission, anterograde trans-synaptic signaling, synapse organization, cellular response to low-density lipoprotein particle stimulus, modulation of chemical synaptic transmission, regulation of positive chemotaxis, and negative regulation of small GTPase mediated signal transduction. Among these, chemical synaptic transmission and anterograde trans-synaptic signaling were also significantly upregulated in oligodendrocytes, in addition to oligodendrocytes’ specific antigen processing and presentation of endogenous peptide antigen via MHC class I via ER pathway, which indicates a possible role in immune signaling and neuroinflammation. OPCs also exhibited mild enrichment in those two antigen related pathways, though this did not reach statistical significance (Fig. 3f). Together, these findings highlight scMINA’s capability to leverage conventional analytical workflows to uncover both cell type-specific and shared functional pathways from multiomic differential expression data, thereby supporting mechanistic interpretation and facilitating hypothesis generation.

### Correlation-based downstream analysis reveals AD-associated shifts and regulatory networks in glial cell types

Beyond conventional downstream analysis, a key feature of the scMINA workflow is its ability to support advanced regulatory inference, enabling users to derive complementary insights and generate more robust hypotheses through data-driven modeling. As previously described, scMINA facilitates refined cell state resolution during integration (Fig. 1b). Here, we demonstrate how scMINA can uncover regulatory signatures even when no fine-grained cell type annotations are available, using microglial population as an example.

Starting with the glial cell population, we first isolated microglial cells, the brain’s primary immune cells, which play a crucial role in AD by transitioning from homeostatic to activated states throughout disease progression [36, 37] and applied scPair to learn a microglia-specific cell state embedding. Since no further subtype annotations were provided, scMINA performed unsupervised Leiden clustering [38] directly on the scPair-derived embeddings and automatically quantified cluster proportions between AD and control conditions. This forms the foundation for both exploratory and condition-specific downstream analysis. In this case study, we identified 11 transcriptionally distinct microglial subpopulations (Fig. 4a). Notably, subclusters 0 and 8 were particularly enriched in AD samples (Fig. 4b), suggesting the presence of an AD-specific microglial state.

**Figure 4.**
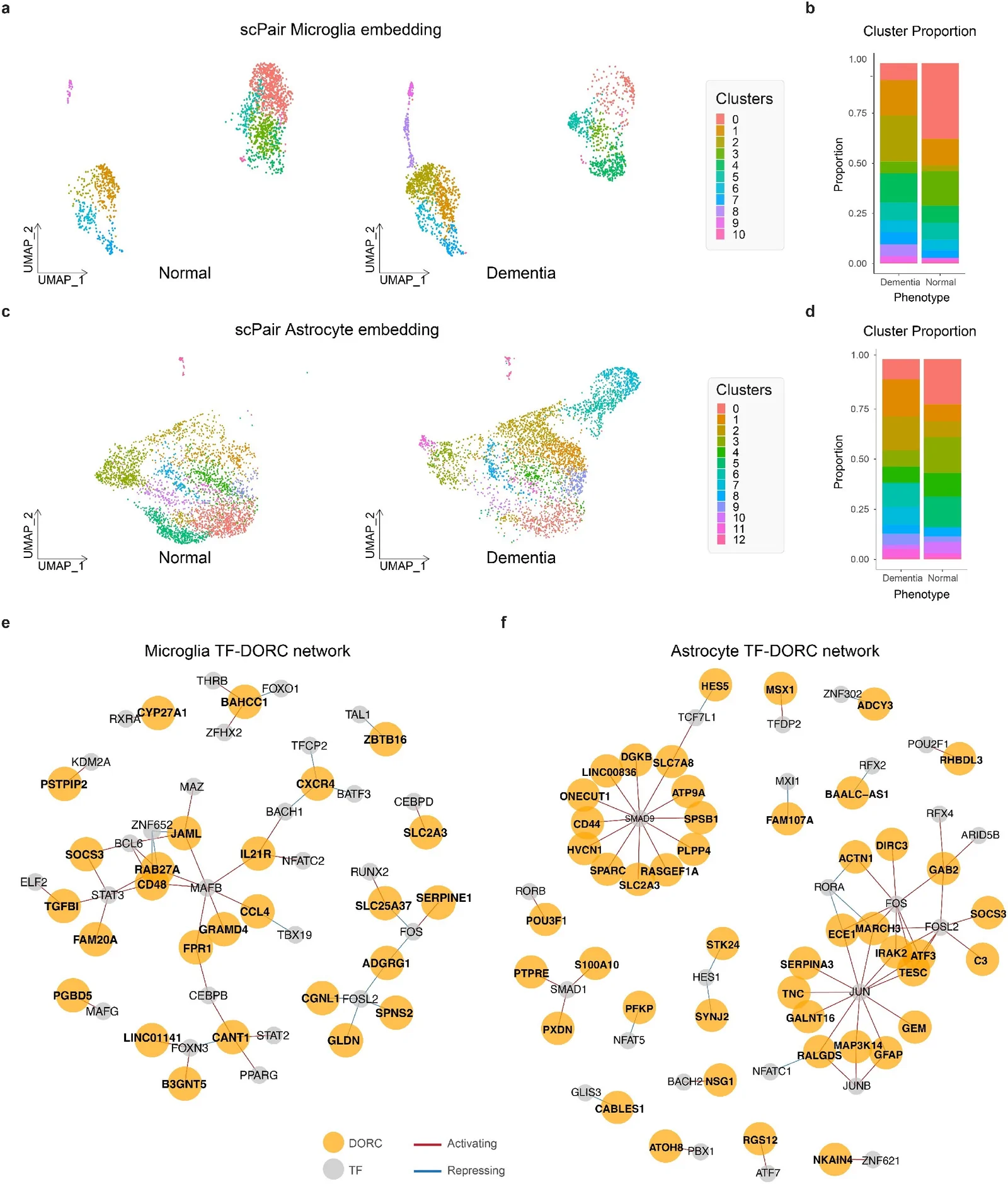
Correlation-based downstream analysis of SEA-AD glial types identifies AD-associated subpopulations and regulatory networks. (**a**) UMAP visualization of microglial cell state space learned by scPair, with each color representing a distinct subpopulation. Left panel: normal control; Right panel: AD case. (**b**) Bar plot comparing the relative abundance of microglial subpopulations (clusters) between normal and AD groups. (**c** and **d**) Similar to (a and b) but for astrocyte. (**e** and **f**) TF-DORC network visualizations for selected DORCs (orange nodes) and their associated TFs (gray nodes) in microglia (e) and astrocyte (f). Edges are scaled and colored by the signed regulation score. Red and blue edges indicate activating and repressive regulation, respectively.

Similarly, scMINA was also performed on astrocytes, which make up the majority of glial population in the human central nervous system. Astrocytes are essential regulators of glutamate and ion homeostasis, cholesterol and sphingolipid metabolism, and they respond dynamically to environmental factors, all of which have been implicated in neurological diseases, including AD [39]. This resulted in the identification of 13 astrocyte subclusters present in both AD and normal samples (Fig. 4c). Among these, subclusters 5, 6, 7, and 11 were enriched in AD donors (Fig. 4d), highlighting potential disease-associated astrocyte states. This unsupervised, cell type-specific subclustering combined with automated group-wise comparison illustrates how scMINA facilitates fine-grained analysis of cellular heterogeneity and disease-driven shifts, even in the absence of additional annotation.

In addition to conventional analyses such as differential gene expression and pathway enrichment (as demonstrated in Fig. 3), scMINA enables correlation-based regulatory analysis through integration with FigR. This module infers TF-target interactions by jointly analyzing gene expression and chromatin accessibility across cell populations. Starting with the microglial population, scMINA computed DORC scores across subclusters and identified 56 high-confidence DORC genes that represent putative regulatory hubs. Similarly, in astrocytes, scMINA identified 164 DORC genes across subclusters, prioritizing features with strong regulatory potential based on multimodal correlations. To further explore upstream regulatory architecture, scMINA used matched TF expression and DORC-linked peak accessibility to construct TF-DORC regulatory networks. In microglia, this network revealed both activating and repressive TF-gene relationships, capturing regulatory programs unique to AD-enriched subpopulations, including the disease-specific cluster 8 (Fig. 4e). Likewise, in astrocytes, the TF-DORC network uncovered condition-specific regulatory relationships that may underlie AD-associated dysfunction, specifically in astrocyte cluster 7 (Fig. 4f).

Together, these results highlight how scMINA’s correlation-based downstream analysis module provides a powerful extension beyond conventional approaches by enabling regulatory inference and disease-specific signature identification. By leveraging matched multimodal data, scMINA identifies both shared and cell state-specific regulatory programs across glial cell types. Importantly, the integration of TF-DORC network construction within this workflow allows users to move from descriptive clustering to mechanistic interpretation, even in the absence of predefined fine-grained annotations.

### Explainable AI-based signature discovery prioritizes key genes in AD-specific cell states

In the AD-enriched microglial subcluster 8, XAI analysis revealed 14 genes with high attribution scores across both methods: *SLC11A2, SMAP2, SYNDIG1, DENND3, NCK2, MRC1, ZBTB16, GLDN, ACSL1, DOCK4, PALD1, RGS1, TMSB4X*, and *SRGN*. Several of these genes have known or emerging links to immune regulation, microglial activation, lipid metabolism, and neurodegenerative disease [40–44]. Among them, four genes—*NCK2, DOCK4, SYNDIG1*, and *GLDN*—also overlapped with DORC features identified in the preceding analysis (Fig. 5a, c, and e). This convergence between XAI-based feature attribution and DORC analysis provides complementary computational evidence supporting their prioritization as candidate features associated with the AD-enriched microglial state.

**Figure 5.**
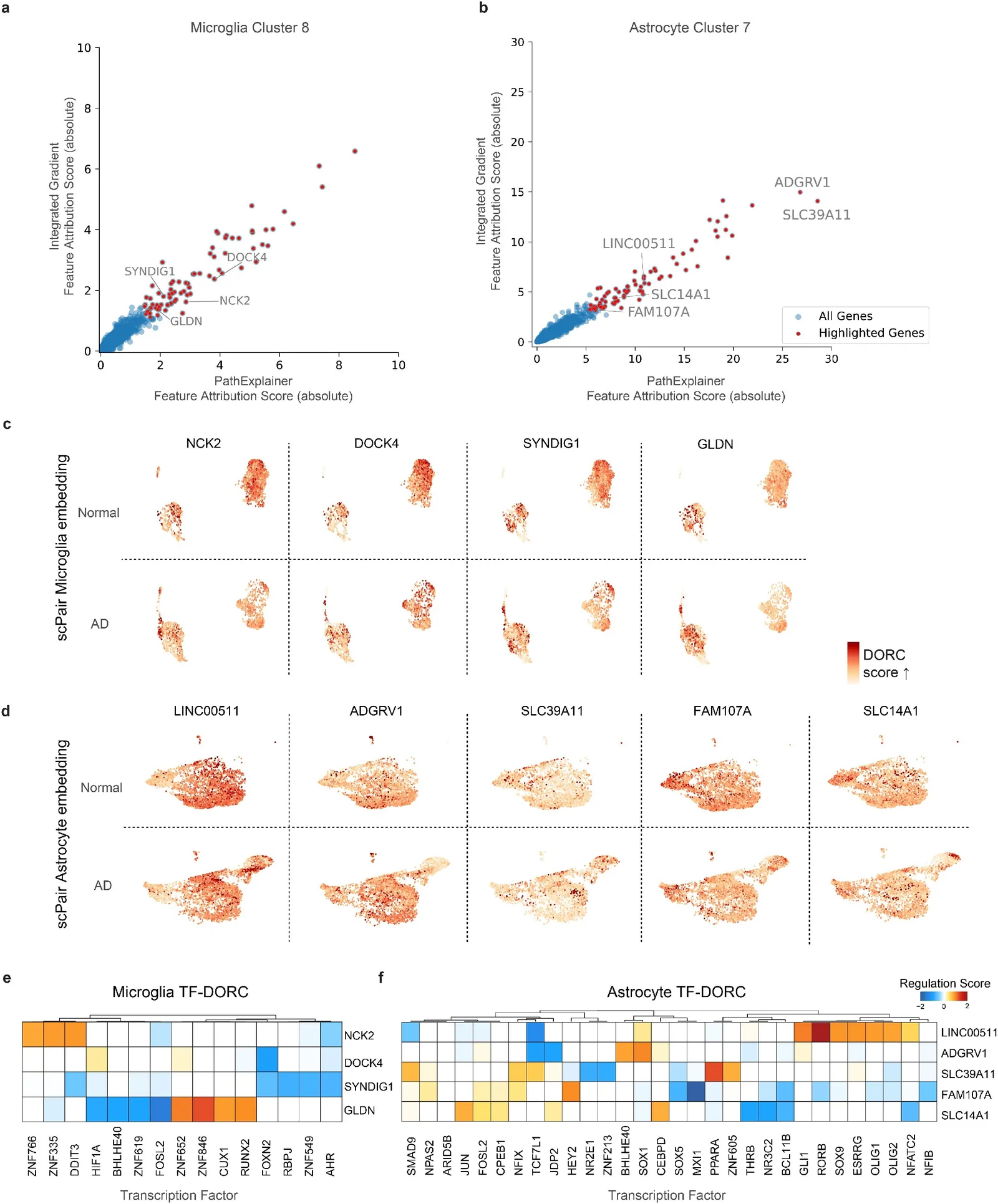
Cross-validation of candidate AD-associated signatures in glial types using explainable AI. (**a** and **b**) Scatterplots showing the absolute feature attribution scores of genes calculated using the Path Explainer (*x*-axis) and Integrated Gradients (*y*-axis) methods on scPair model for corresponding cluster(s). (a) The red-colored genes represent the intersection of the top 100 genes identified by both methods within cluster 8 of microglia. (b) The red-colored genes represent the intersection of the top 100 genes identified by both methods within cluster 7 of astrocyte. The displayed gene names are representatives of highly attributed genes intersecting with identified DORC genes. (**c** and **d**) UMAP visualization of microglia (c) and astrocyte (d) cell state space learned by scPair, split by selected intersecting genes and disease status. The color gradient represents the DORC score for each cell. Left: normal control. Right: AD case. (**e**) Heatmap of DORC regulation scores showing significant TF-DORC enrichments for selected DORC genes (*NCK2, DOCK4, SYNDIG1*, and *GLDN*) in microglia cluster 8. The heatmap is colored by regulation scores. (**f**) Similar to (e) but for selected DORC genes (*LINC00511, ADGRV1, SLC39A11, FAM107A, and SLC14A1*) in astrocyte cluster 7.

These candidate signatures were further contextualized using scMINA’s correlation-based TF–DORC network. Within subcluster 8, the analysis prioritized several transcription factors, including *ZNF766, ZNF335*, and *ZNF846*. Several members of the broader zinc-finger transcription factor family have previously been implicated in AD-associated molecular processes, providing potential biological context for the ZNF-family candidates prioritized here [45]. The analysis also prioritized *HIF-1α*, a hypoxia-responsive transcription factor, as a candidate factor associated with microglial and neuroinflammatory regulatory programs in AD (Fig. 5e). Previous studies have reported context-dependent protective and pathological roles for *HIF-1α* in AD-related processes, including modulation of tau phosphorylation and inflammatory signaling [46]. Together, these computational and literature-supported observations nominate *HIF-1α* and the identified ZNF-family transcription factors as candidates for further investigation; however, their causal regulatory roles in these microglial states remain to be established experimentally.

In parallel, we applied the same interpretability analysis to astrocytes, focusing on subclusters 5, 6, 7, and 11, which were enriched in AD samples. This analysis identified 15 genes that were both highly ranked by the XAI methods and classified as DORCs: *GFAP, LINC00511, TNC, SLC39A11, ADGRV1, CABLES1, FAM107A, DCLK1, LRIG1, HPSE2, CD44, SLC14A1, GALNT15, SPARCL1*, and *SERPINA3*. Within subcluster 7, for example, *ADGRV1, SLC39A11, SLC14A1, FAM107A*, and *LINC00511* emerged as high-priority candidate features associated with the AD-enriched astrocyte state (Fig. 5b, d, and f). Several of these genes have previously been implicated in astrocyte biology or AD-associated astrocyte transcriptional changes, including *SLC39A11* and *SLC14A1* in human AD astrocytes and *ADGRV1* in astrocyte homeostasis and glutamate regulation [47–49]. Collectively, these genes span diverse biological functions, suggesting that multiple molecular processes may contribute to AD-associated astrocyte states.

The TF–DORC analysis additionally prioritized candidate transcription factors associated with these astrocyte states. Among these were *JUN* and *FOSL2*, components of the activator protein 1 (AP-1) complex that have previously been associated with neuroinflammatory signaling and astrocyte reactivity. Disease-associated astrocyte states have been identified in AD and aging [50], with subsequent chromatin accessibility and transcriptomic analyses implicating *FOSL2* as a potential regulator of disease-associated astrocyte transcriptional programs [51]. *PPARA* was also prioritized by our analysis. *PPARA* is involved in transcriptional regulation of lipid and fatty-acid metabolism, mitochondrial function, and other cellular processes relevant to neuronal and glial homeostasis [52]. Previous experimental studies have further linked PPARA signaling to amyloid-related pathology; for example, administration of PPARA agonists has been reported to reduce amyloid pathology and improve behavioral phenotypes in APP–PSEN1 mouse models [53]. These prior observations provide biological context for the candidates prioritized by scMINA but do not, by themselves, establish their regulatory roles in the specific AD-associated astrocyte states identified here.

Collectively, these analyses illustrate the ability of scMINA to prioritize candidate disease-associated genes and transcription factors by integrating complementary information from cell-state embeddings, DORC-based regulatory inference, and XAI-based feature attribution. Concordance across these computational analyses, together with support from previously reported biological observations, provides a basis for generating testable hypotheses regarding molecular programs associated with AD-related glial states. Importantly, these findings should be interpreted as candidate regulatory associations rather than evidence of causal regulation. Experimental perturbation and orthogonal validation will be required to establish the functional roles of these prioritized genes and transcription factors in AD pathogenesis.

Finally, to facilitate interpretation and downstream exploration, scMINA provides both visual and tabular summaries of prioritized features. These include: (i) scatter plots comparing attribution scores from Integrated Gradients and Path Explainer, with concordantly high-ranking genes highlighted; (ii) UMAP visualizations with gene-specific DORC score overlays to illustrate cell-level molecular patterns; and (iii) downloadable tables linking high-attribution features to regulatory annotations, including DORC status, and inferred TF–gene associations. These outputs enable users to explore and interpret computationally prioritized candidate targets and to nominate features for subsequent biological and experimental validation.

## Discussion

In this study, we introduced scMINA, an interpretable and modular workflow for integrative analysis of single-cell multiomic data. scMINA addresses key challenges in preprocessing, modality alignment, and downstream interpretation of multiome datasets, while offering a flexible design that accommodates diverse inputs and analysis goals. A central innovation of scMINA is its combination of advanced integration strategies with multiple layers of downstream analysis, including both correlation-based and XAI methods for mechanistic insight and hypothesis generation.

At its core, scMINA is designed for flexibility and extensibility. Users can initiate analysis from raw fragments and count matrices or enter at later stages using pre-processed or annotated data. The integration module is modular and supports benchmarking of multiple methods. Among them, scPair is designated as the default due to its strong performance in supervised multimodal representation learning, offering robust alignment between chromatin accessibility and gene expression modalities.

A major contribution of scMINA is the incorporation of explainable AI techniques to address one of the most persistent challenges in deep learning: the interpretability of learned embeddings. Deep neural networks often capture complex biological structure but lack transparency in their internal representations. scMINA addresses this by incorporating feature attribution methods, such as Integrated Gradients and Path Explainer, to identify input features (e.g. genes or peaks) most influential in shaping cell state embeddings. These attribution-derived signatures provide a direct link between model outputs and biological drivers. To increase robustness, scMINA also supports correlation-based regulatory inference via FigR, which links transcription factor expression to chromatin accessibility at putative regulatory domains. Together, these three layers, conventional, correlation-based regulatory analysis, and XAI-based attribution, enable cross-validation of candidate genes and regulators, improving confidence in prioritized signatures and enhancing biological interpretability. This multi-layered design makes scMINA especially valuable for disease mechanism studies, regulatory network reconstruction, and therapeutic target discovery.

We demonstrated the utility of scMINA using a large paired single-cell multiome dataset from the SEA-AD consortium. The workflow recapitulated previously reported AD-associated transcriptional and regulatory signatures while also prioritizing candidate genes and transcription factors associated with disease-enriched cellular states. By integrating evidence from multimodal cell-state representations, DORC-based regulatory inference, and XAI-based feature attribution, scMINA provides complementary computational perspectives for prioritizing molecular features and generating biologically informed, experimentally testable hypotheses. Importantly, these prioritized features should be interpreted as candidate regulatory associations rather than established causal regulators, and their functional roles will require validation through independent experimental and perturbation-based studies.

Although demonstrated here in the context of neurodegenerative disease, the analytical design of scMINA is not inherently restricted to a particular tissue, disease, or species and may therefore be adapted to other paired single-cell multiome datasets. Its modular architecture allows users to substitute core components, including integration models, clustering approaches, and attribution methods, according to dataset characteristics and analytical objectives. For example, although scPair is implemented as the default integration option in the current workflow, alternative integration approaches can be incorporated, and previously integrated and annotated datasets can enter the workflow at downstream stages. Intermediate outputs, including learned embeddings, DORC matrices, cluster annotations, and ranked feature tables, are retained and made accessible to facilitate iterative analyses, transparent inspection of intermediate results, and integration with external tools or prior biological knowledge.

Several limitations should be considered when interpreting the present study. First, the biological application and downstream regulatory prioritization were demonstrated primarily using the SEA-AD dataset, and evaluation across additional disease contexts and multiome datasets will be valuable for further assessing the generalizability of the overall scMINA workflow. Second, the concordance observed among XAI-based attribution, DORC analysis, and TF–DORC network inference represents convergent computational evidence rather than independent experimental validation. In particular, the candidate genes and transcription factors prioritized in AD-associated microglial and astrocyte states have not been functionally validated in the present study. Future perturbation-based and *in vivo* studies will therefore be important for determining whether these candidates have causal roles in disease-associated cellular phenotypes.

As single-cell multiomic technologies continue to increase in scale and complexity, computational frameworks that integrate multimodal information while maintaining interpretability will be increasingly important for extracting testable biological hypotheses from high-dimensional data. By combining multimodal integration, correlation-based regulatory inference, and interpretable machine learning approaches within a modular workflow, scMINA provides a practical strategy for characterizing cellular states and prioritizing candidate regulatory features from paired single-cell multiome data. Rather than establishing causal regulatory relationships, scMINA is designed to narrow the space of candidate genes and transcription factors for subsequent biological investigation, thereby providing a bridge between multimodal computational analysis and hypothesis-driven experimental studies.

## Data Availability

The multiome data used in this study can be accessed from AD knowledge Portal under ID: syn29879329: https://www.synapse.org/Synapse:syn29879329. The scMINA framework and analysis scripts are publicly available at 10.5281/zenodo.19582768 and https://github.com/ShuwenZhangswz/scMINA, including documentation and example workflows for reproducing the analyses described in this study.
